# Correlation Between Oxygen Desaturation Index Measured by Overnight Oximetry and Apnea-Hypopnea Index Measured by Polysomnography in Patients Diagnosed With Obstructive Sleep Apnea

**DOI:** 10.7759/cureus.71895

**Published:** 2024-10-19

**Authors:** Asha Undrajavarapu, Arun Prasath, Mathew Varghese, Prince George, King Herald Kisku

**Affiliations:** 1 Pulmonary Medicine, All India Institute of Medical Sciences, Bhopal, Bhopal, IND; 2 Respiratory Medicine, Pondicherry Institute of Medical Sciences, Puducherry, IND; 3 Respiratory Medicine, Welcare Hospital, Kochi, IND; 4 Respiratory Medicine, Lakshmi Hospital, Kochi, IND; 5 Pulmonary Medicine, Al-Amiri Hospital, Ministry of Health, Kuwait City, KWT

**Keywords:** apnea-hypopnea index (ahi), obstructive sleep apnea (osa), oxygen desaturation index (odi), polysomnography (psg), sleep disordered breathing (sdb)

## Abstract

Background

The most common sleep-disordered breathing (SBD) is obstructive sleep apnea (OSA), which affects nearly a billion adults worldwide. OSA is characterized by frequent episodes of a complete (apnea) or partial (hypopnea) collapse of the upper airway, mainly the oropharyngeal tract during sleep, with a consequent cessation or reduction of the airflow. Undiagnosed and untreated OSA can lead to cognitive and neurobehavioral dysfunction, cardiovascular comorbidities, inability to concentrate, memory impairment, and mood changes like irritability and depression, with a remarkable effect on quality of life. Polysomnography (PSG) remains the gold standard for diagnosis; however, it is expensive, less accessible, and has a long waiting period to get it done. Home nocturnal oximetry, measuring the oxygen desaturation index (ODI), is a promising alternative for diagnosis.

Materials and methods

A prospective observational study was conducted over two years, involving patients with a high pre-test probability of OSA. Patients underwent both home nocturnal oximetry and in-lab PSG. Statistical analysis was performed using the Spearman correlation, the Wilcoxon signed-rank test, and the receiver operating characteristic (ROC) curves to compare ODI and apnea-hypopnea index (AHI) values.

Results

Out of 67 participants, 43 (64%) had abnormal home oximetry results (ODI>5) and 62 (92.5%) had abnormal PSG (AHI>5). There was a significant correlation between ODI and AHI (r=0.734, p=0.001). No significant difference was found between ODI values from home oximetry and in-lab PSG. The sensitivity, specificity, and diagnostic accuracy of home oximetry in detecting OSA (ODI>5) were 69%, 100%, and 71%, respectively. The ROC analysis demonstrated an area under the curve (AUC) of 0.98 for predicting AHI>5.

Conclusion

Home nocturnal oximetry correlates well with level 1 PSG and provides a reliable diagnostic tool for OSA, especially in resource-limited settings. However, further validation is required to improve its sensitivity when ruling out OSA.

## Introduction

Obstructive sleep apnea (OSA) is the most prevalent sleep-disordered breathing (SDB), and the global prevalence is estimated to be one billion in adults aged 30-69 years worldwide. The spectrum ranges from mild to severe OSA, and a total of 425 million adults were approximately reported to have moderate-to-severe OSA. Among Indians, 9.6% are known to have OSA and 5.4% have moderate-to-severe OSA [1].

The etiology of OSA is multifactorial consisting of the interplay between anatomic and neuromuscular factors and an underlying genetic predisposition toward the disease. Risk factors include snoring, male gender, age, menopause in women, obesity, and anatomical abnormalities such as craniofacial and oropharyngeal features, including retro or micrognathia, nasal obstruction, enlarged tonsils/adenoids, macroglossia, and low-lying soft palate [2,3]. Daytime sleepiness, due to nocturnal sleep fragmentation, is a key symptom of OSA, being present in more than 80% of the patients.

The diagnosis of OSA is traditionally by a full-night polysomnography (PSG) study performed in a sleep laboratory. PSG is a comprehensive recording of the neurophysiological and cardiorespiratory changes that occur during sleep [4]. However, it is relatively expensive and has a critical wait time, which has led to the design of simpler portable devices. Home oximetry has been proposed as a reliable and valuable screening tool in these settings to detect and treat clinically significant OSA. The advantages of portable devices are that they can be used in outpatient settings and large groups of patients can have access to testing and treatment [4,5]. Oxygen desaturation obtained from nocturnal pulse oximetry is a significant evaluation parameter for sleep-related breathing disorders, especially for the diagnosis of OSA. Clinical interpretation relies mainly on the number of desaturation events per hour, known as the oxygen desaturation index (ODI). This parameter has been validated in various studies [5].

This study aims to examine the sensitivity, specificity, positive predictive value, and negative predictive value of overnight oximetry in the diagnosis of OSA as defined by level 1 PSG to correlate ODI obtained by overnight oximetry and apnea-hypopnea index (AHI) obtained by PSG done in the sleep lab and to compare nocturnal oximetry readings done at home and in the sleep lab.

This article was previously presented as a meeting abstract at the Virtual National Conference on Pulmonary Diseases (NAPCON) 2020 held from January 27-31, 2021. 

## Materials and methods

This was a prospective observational study conducted over a period of two years (November 2018 to November 2020) in the Department of Pulmonary Medicine, Sleep Lab, and Department of Physiology. The study was commenced after approval was obtained from the Institutional Ethics Committee (IEC: RC/18/90). This study included patients with an Epworth sleepiness scale (ESS) score >10 and a high clinical probability of OSA as evidenced by the snoring, tiredness, observed apnea, high blood pressure, body mass index, age, neck circumference, and male gender (STOP-Bang) score (see Table 7 in Appendices) with age >18 years. Those patients with pre-existing chronic respiratory failure with peripheral oxygen saturation (SpO_2_) <90% and acutely ill patients were excluded.

A few terms used in this study are defined by the American Academy of Sleep Medicine (AASM), 2014 as follows: Apnea is defined as the flow reduction up to ≥90% for >10 seconds; Hypopnea is defined as the flow reduction up to ≥30% for >10 seconds and ≥4% desaturation; ODI is defined as the average number of desaturation episodes per hour of recording; AHI is defined as the number of apnea and hypopnea events per hour of sleep.

**Figure 1:**
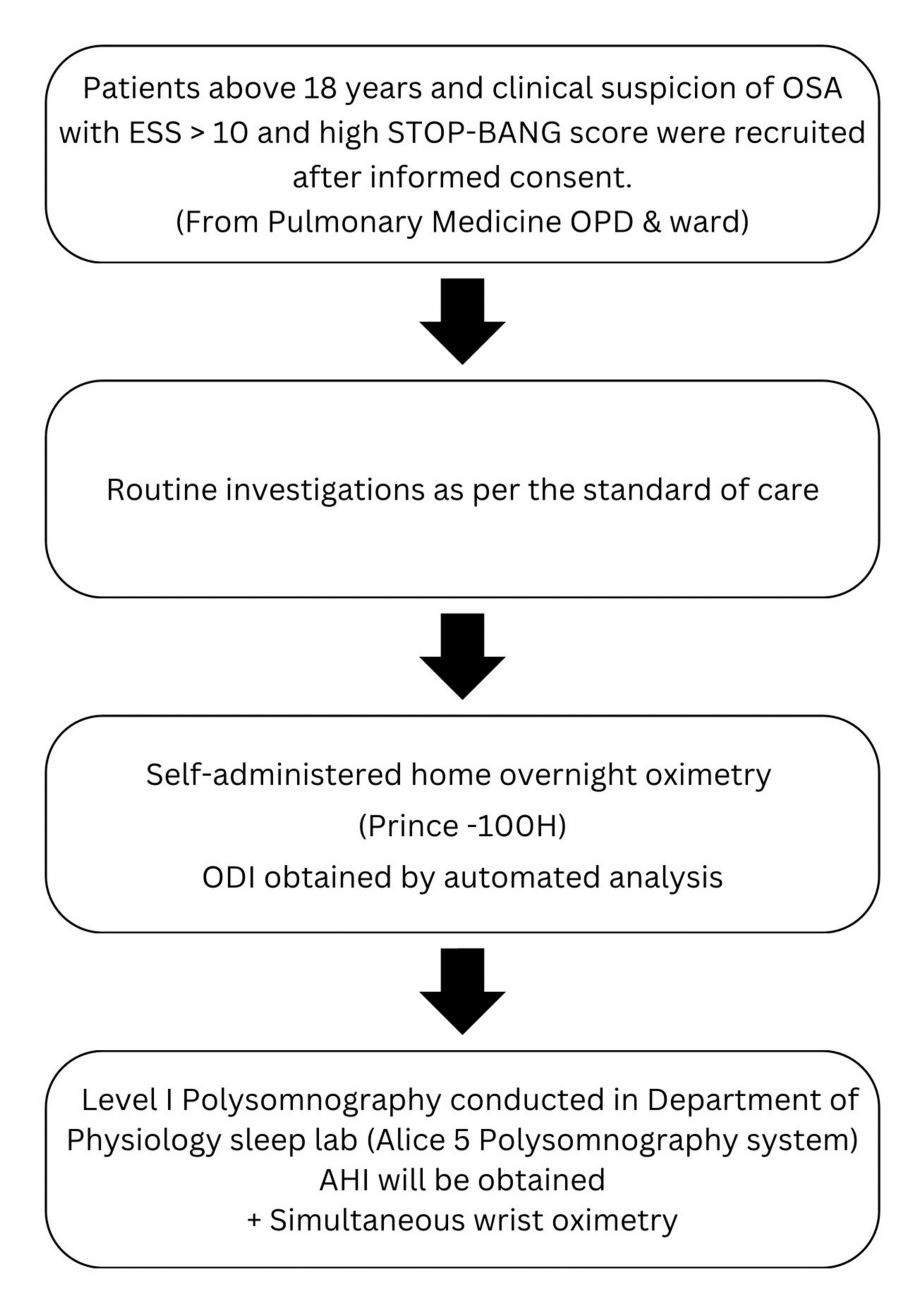
STROBE ESS: Epworth sleepiness scale, ODI: Oxygen desaturation index, AHI: Apnea-hypopnea index; STROBE: Strengthening the Reporting of Observational Studies in Epidemiology; STOP-Bang: Snoring, tiredness, observed apnea, high blood pressure, body mass index, age, neck circumference, and male gender

Assuming a sensitivity of 80%, precision of 9%, and a desired confidence level of 95%, the sample size was estimated to be 79. However, due to the restrictions imposed by the COVID-19 pandemic, after discussion with and due approval acquired from the research and ethics committees, the study population was modified to 67 patients. A total of 67 patients who fulfilled the inclusion criteria were evaluated and informed consent was obtained. The clinical parameters recorded included a detailed clinical history focusing on the risk factors of OSA such as hypertension, dyslipidemia, history of cardiovascular disorders, presence of comorbid conditions, occupational history, and clinical variables including the severity of dyspnea, history of smoking, anthropometric measurements like body weight, height, body mass index (BMI), and neck circumference and other signs clinically associated with OSA. Screening questionnaires including STOP-Bang and ESS were used (Figure 1).

Continuous variables like age, anthropometric measurements (weight, height, BMI), AHI, and ODI were reported as mean and standard deviation. Categorical variables like gender and the results of the screening questionnaires were analyzed and represented as frequencies and percentages. Data were entered into Microsoft Excel software (Microsoft Corp., Redmond, USA) and were analyzed using IBM SPSS Statistics for Windows, version 17.0 (IBM Corp., Armonk, USA) and GraphPad Prism 9 (Dotmatics, Boston, USA). Spearman correlation was used to establish the correlation between home and in-lab oximetry and between ODI values and AHI values. The Wilcoxon paired signed-rank sum test was used to compare the difference between home and in-lab ODI values, which had a non-gaussian distribution. The sensitivity, specificity, positive predictive value, and negative predictive value were estimated and are reported in the results. The receiver operating characteristic (ROC) curve was constructed, and the area under the curve (AUC) was calculated to determine sensitivity characteristics assuming varying cut-off levels for ODI as compared to the gold standard, i.e., AHI. A p-value <0.05 was considered statistically significant for all comparative statistics.

## Results

A total of 67 patients fulfilling the inclusion criteria were a part of the final study analysis. Out of these, 44 (66%) were males while 23 (34%) were females. The mean age of our study population was 50.32 ± 13 years (range 21-81 years). A majority of the study population belonged to the 50-60 years age group. Table 1 shows the demographics and comorbidities of the patients included in the study.

**Table 1:** Demographic characteristics of the study population The values are presented as N (%) and mean ± SD.

Patient characteristics	N (%)
Age	
Male	44 (66%)
Female	23 (34%)
Mean age = 50.32 ± 13	
Comorbidities	
Systemic hypertension	40 (59.70%)
Diabetes mellitus	19 (28.35%)
Thyroid disease	5 (7.46%)
Obstructive airway disease	7 (10.44%)
Coronary artery disease	3 (4.47%)
Smoking history	
Present	9 (13.43%)
Absent	58 (86.57%)

The average BMI of our study population was 34.75 ± 6.49 kg/m^2^. Furthermore, a frequency distribution of BMI among our study population showed that 32.8% of the study population had class I obesity followed by class II obesity in 26.9% of the population.

Around 3% of the study population had normal BMI. In the study population, 58 (86.56%) participants had an ESS score ≥10, and nine (13.43%) participants had an ESS score <10. With regards to the STOP-Bang score, 41 (61.19%) participants had a STOP-Bang score in the high-risk (score >5-8) category, while 26 (38.80%) participants had in the intermediate-risk (score 3-4) category. The mean STOP-Bang score was 5.29±1.40, while the mean ESS was 15.1 ± 4.68. The mean AHI of the study population, as determined by overnight diagnostic PSG, was 46.40±33.73 events per hour. As many as 62 (92.5%) participants had abnormal studies indicated by AHI>5 (Table 2). Some 24 (35.8%) participants had normal studies as indicated by ODI<5 and 43 (64%) had abnormal studies indicated by ODI>5. Among them, there were 18 (26.8%) who had mild OSA (5≤ODI≤15), 20 (29.85%) had moderate OSA (15<ODI≤30), and five (7.46%) had severe OSA (ODI>30) (Table 3).

**Table 2:** AHI obtained by Polysomnography The values are presented as N (%). AHI: Apnea-hypopnea index; OSA: Obstructive sleep apnea

Severity	N (%)
Normal: AHI<5	5 (7.46)
Mild OSA (5≤AHI≤15)	8 (11.94)
Moderate OSA (>15)	17 (25.37)
Severe OSA (>30)	37 (55.22)

**Table 3:** ODI obtained by home overnight oximetry The values are presented as N (%). ODI: Oxygen desaturation index; OSA: Obstructive sleep apnea

Severity	N (%)
Normal: ODI<5	24 (35.82)
Mild OSA (5≤ODI≤15)	18 (26.86)
Moderate OSA (>15)	20 (29.85)
Severe OSA (ODI>30)	5 (7.46)

**Figure 2:**
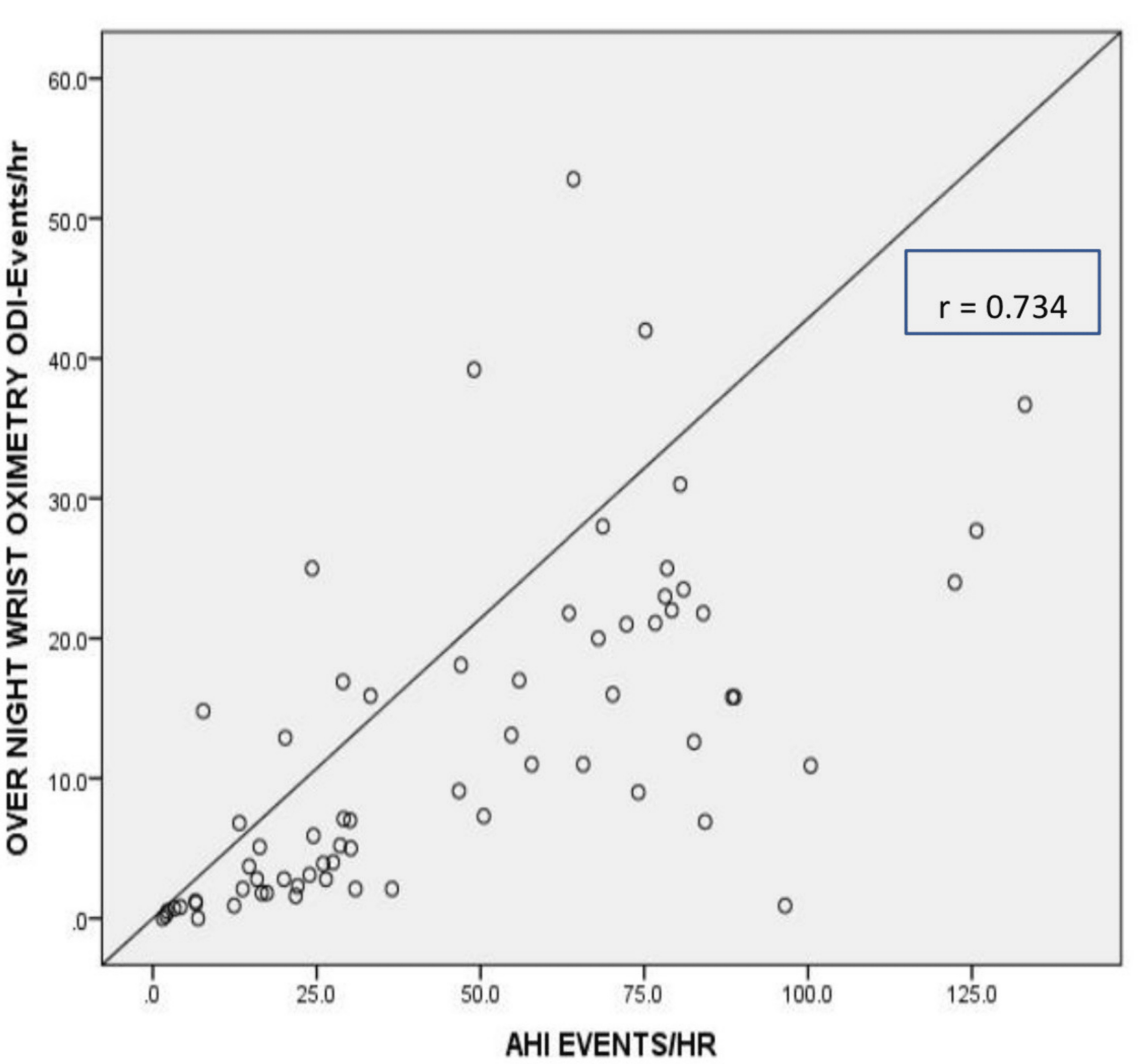
Scatter plot showing correlation between ODI and AHI ODI: Oxygen desaturation index; AHI: Apnea-hypopnea index

The study participants underwent nocturnal oximetry at home and on consecutive days in the sleep lab along with simultaneous diagnostic PSG. The mean ODI of nocturnal oximetry done at home was 12.31 with a standard deviation of 11.70. The mean ODI of nocturnal oximetry done in the sleep lab, along with simultaneous diagnostic PSG, was 12.09 with a standard deviation of 11.52. The ODI values obtained at home and in the laboratory were found to correlate well (r=0.9825, p<0.0001). Considering the non-gaussian distribution of the values, the differences between the median values of the two sets of ODI values were analyzed by the Wilcoxon matched-pair signed-rank test (Table 4). There was no statistically significant difference between the ODI values obtained from home oximetry and those done in the sleep laboratory (p>0.05). There was a linear correlation between ODI assessed by home overnight oximetry and AHI measured by the PSG. This correlation was found to be statistically significant (r=0.734, p=0.001) (Figure 2).

**Table 4:** Comparison of nocturnal oximetry readings done at home and in the sleep lab The values are presented as mean ± SD, median, and interquartile range. P-value (<0.05, 0.001) is considered significant. ODI: Oxygen desaturation index

	Home - Nocturnal Oximetry ODI	Sleep Lab - Nocturnal Oximetry ODI
Mean ± SD	12.31 ± 11.70	12.09 ± 11.52
Median	9	10.1
Interquartile range	2.55 - 20.5	2.4 - 18.55
Wilcoxon test	p=0.4237	

Assuming a cut-off of ODI≥5 to designate as diagnostic for OSA, predictive validity of overnight oximetry in predicting AHI≥5 (cut-off for diagnosis of mild OSA) was calculated (Table 5). The sensitivity was found to be 0.69, specificity 1.00, positive predictive value 1.00, negative predictive value 0.20, and the diagnostic accuracy was 0.71. Using a cut-off of ODI≥15 designated as diagnostic for moderate to severe OSA, the predictive validity of overnight oximetry in predicting AHI≥15 (standard for diagnosing moderate to severe OSA) was calculated (Table 6). The sensitivity was determined to be 0.46, specificity 1.00, positive predictive value 1.00, negative predictive value 0.30, and the diagnostic accuracy was 0.56.

**Table 5:** Predictive validity of ODI≥5 obtained by overnight oximetry in predicting AHI≥5 by diagnostic polysomnography ODI: Oxygen desaturation index; AHI: Apnea-hypopnea index

Parameter	Value
Sensitivity	0.69
Specificity	1.00
Positive predictive value	1.00
Negative predictive value	0.20
Diagnostic accuracy	0.71

**Table 6:** Predictive validity of ODI≥15 obtained by overnight oximetry in moderate-to-severe OSA by diagnostic polysomnography ODI: Oxygen desaturation index; AHI: Apnea-hypopnea index

Parameter	Value
Sensitivity	0.46
Specificity	1.00
Positive predictive value	1.00
Negative predictive value	0.30
Diagnostic accuracy	0.56

**Figure 3:**
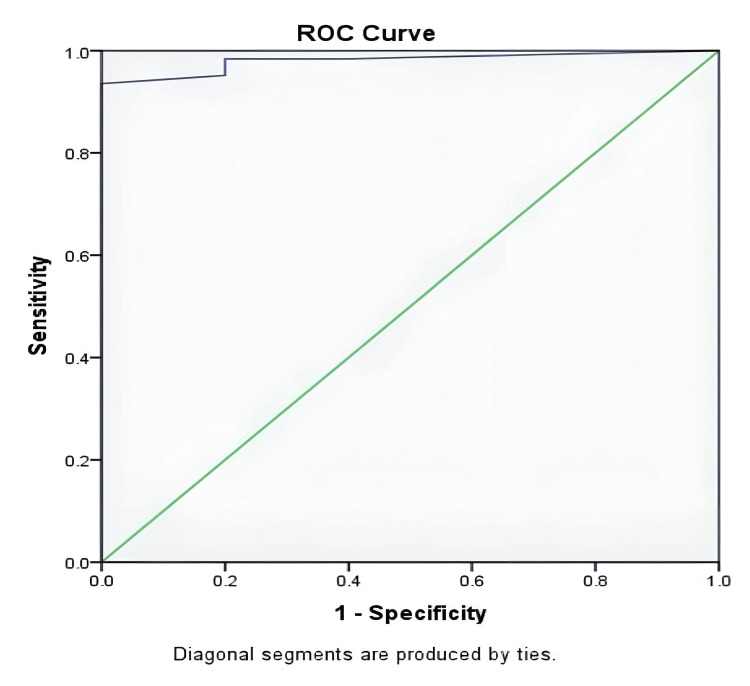
Sensitivity and specificity based on AUROC ROC: Receiver operating characteristic curve; AUROC: Area under the ROC curve

**Figure 4:**
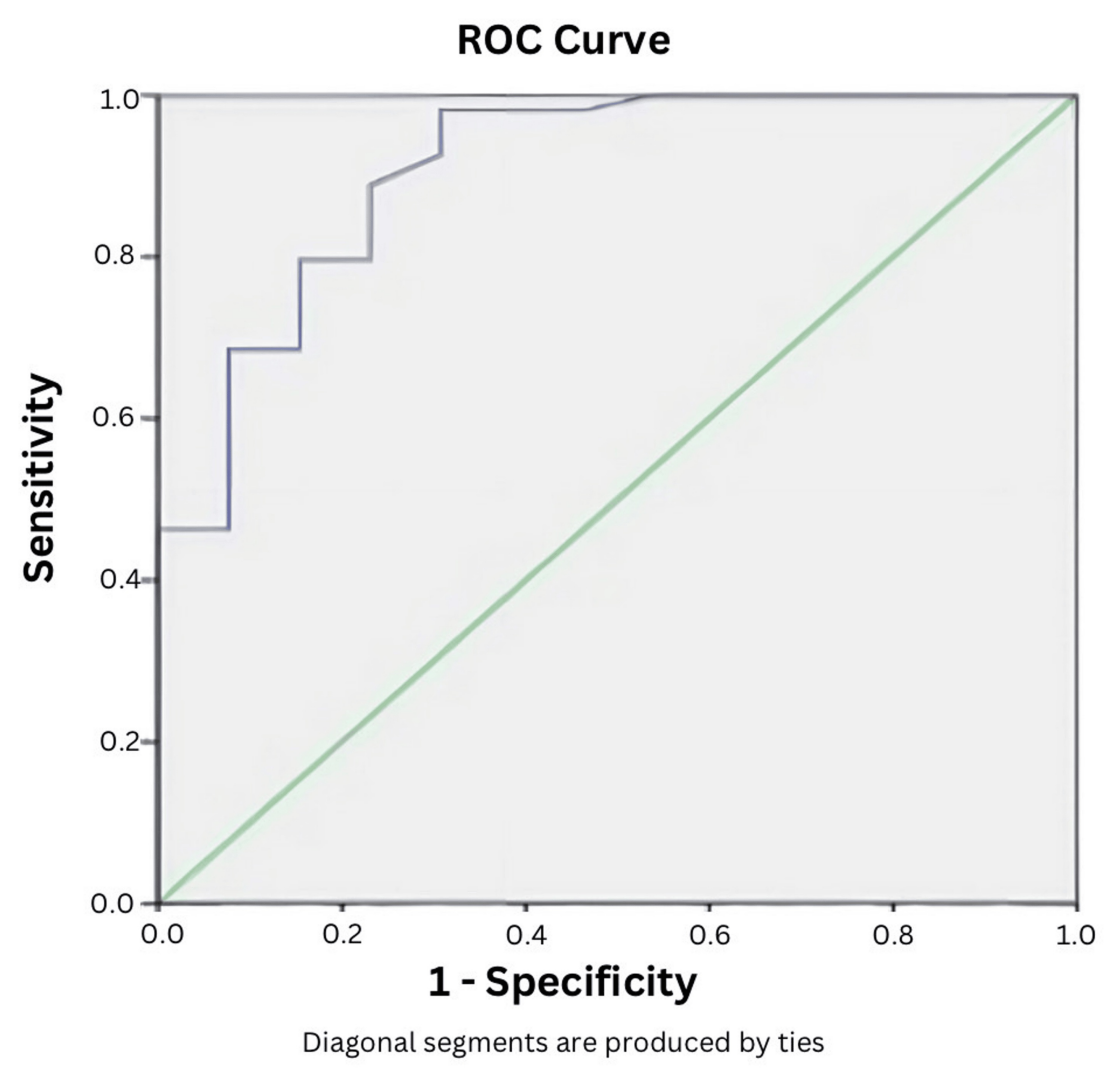
Sensitivity and specificity based on AUROC (moderate-to-severe OSA) ROC: Receiver operating characteristic curve; AUROC: Area under the ROC curve; OSA: Obstructive sleep apnea

AUC for home overnight oximetry in detecting AHI>5 was found to be 0.98, and the lower and upper limits were 0.94 and 1.00, respectively. The optimal value of ODI obtained from ROC was 1.2, from which sensitivity was 0.93, specificity was 1.00, positive predictive value was 1.00, and negative predictive value was 0.55, with a diagnostic accuracy of 0.94 (Figure 3). AUC for home overnight oximetry in detecting AHI>15 was determined to be 0.90, and the lower and upper limits were 0.81 and 1.00, respectively. The optimal value of ODI obtained from ROC was 1.4, from which the sensitivity was 0.98, specificity was 0.69, positive predictive value was 0.93, and negative predictive value was 0.90, with a diagnostic accuracy of 0.92 (Figure 4).

## Discussion

OSA is the most common sleep-related breathing disorder, with a prevalence of up to 19.5% in the Indian urban male population [6]. Up to 82% of males and 93% of females remain undiagnosed [7]. The complications associated with OSA are cardiovascular morbidities, cognitive impairment, diabetes mellitus, and premature death.

PSG is the gold standard for the diagnosis of OSA. A full night diagnostic sleep study in a level 1 full montage set-up is certainly the ideal way to diagnose and manage OSA. However, such set-ups are costly as well as limited to a few sleep laboratories. Portable devices are useful alternatives to the standard diagnostic PSG, which has the added advantage of being conducted in the home environment. There is a growing tendency to use simplified monitored devices not only in developing countries but also in developed ones. The technical advancements in portable monitors have led to the widespread screening of OSA. Type 4 monitors such as nocturnal pulse oximetry have been indicated for screening of OSA [8]. Oximetry-based variables like ODI can be used in the diagnostic evaluation of OSA. Nevertheless, there is a need to document the diagnostic efficacy of a type 4 monitoring device as compared to that of a level 1 set-up.

The present study was conducted to determine the predictive value of overnight oximetry in OSA. We compared the ODI by nocturnal pulse oximetry done at home with AHI values obtained by type 1 diagnostic PSG.

The mean age of the study population was 50.32 ± 13 years. The majority of the patients belonged to the 51-60 years age group. In a study published by Kendzerska et al. [9] in 2014, similar age and gender characteristics were observed. In the 10,149 patients finally included in their study, the mean age was 50 years and male patients constituted 62% of the study population. The mean BMI was 34.75 ± 6.49 kg/m^2^, and the majority belonged to class I obesity. Pataka et al. [10] conducted a study on 1,853 subjects, and the mean BMI of the study population was 32.8 ± 7 kg/m^2^. BMI is a significant risk factor for OSA. An increase in BMI has been shown as an independent predictor of OSA and a greater oxygen desaturation during sleep, especially in the recumbent position [11].

From level 1 PSG conducted in all our patients, the mean AHI of the study population was 46.40 ± 33.73 events per hour. Around 7.46% had normal AHI and 92.64 % had positive AHI (i.e., ≥5 events/hour). Using PSG AHI of 5 as the cut-off value, the prevalence of OSA in the study population was 92.64%. AHI≥30 was found in 55.2%, 15<AHI≤30 in 25.3%, 5≤AHI≤15 in 11.9%, and AHI<5 in 7.4% participants, respectively. Chung et al. [12] analyzed 516 patients, of whom 10% were found to be non-OSA, 38.9% mild OSA, 16.7% moderate OSA, and 16.7% severe OSA. From the overnight oximetry, the mean ODI of the study population was 12.3 ± 11.7. About 35.8% of the participants had normal study as indicated by ODI<5, and 64% had abnormal study indicated by ODI>5. About 26.8% had mild OSA (5≤ODI≤15), 29.85% had moderate OSA (15<ODI≤30), and 7.46% had severe OSA (ODI>30).

The “first night effect” is a well-known phenomenon, when a patient is made to sleep in an alien environment and it affects the majority of sleep parameters. We intended to analyze the ODI variability in patients’ own environment with that done in an artificial laboratory setting. The median values and interquartile ranges were calculated for the ODI values obtained from home oximetry and those acquired from the in-lab measurements. The non-parametric Wilcoxon signed-rank sum test was applied, and the difference in medians between the home oximetry ODI values and the in-lab values were found to be statistically not different (p>0.05). The findings of our study implicate minimal and therefore acceptable, intra-individual night-to-night variability of ODI, despite a change in environment. Hence, the diagnostic evaluation can reliably be done on a single night measurement. A similar study conducted by Fietze et al. [13] in 2004 on 35 patients with mild-to-moderate OSA also reported minimal night-to-night variability in ODI and concluded that it is reliable to depend on the results of a single one-night sleep study recording for the diagnosis of OSA. On the other hand, a study by Stöberl et al. [14] in 2017 reported considerable night-to-night variation of ODI in patients of OSA taken off their prescribed continuous positive airway pressure (CPAP) therapy.

In this study, we attempted to evaluate the correlation between the ODI values obtained from home oximetry and the AHI values obtained from PSG. The two sets of parameters were found to correlate well with each other (r=0.734, p=0.001). A similar strong correlation between ODI and AHI has been reported by Nair et al. in their series [15].

The ability of overnight home oximetry to effectively diagnose OSA was determined by comparing with the accepted gold standard, i.e., AHI obtained from PSG at two levels: AHI≥5 events/hour compared to ODI≥5 for the diagnosis of OSA, and AHI≥15 events/hour compared to ODI≥15 for diagnosing moderate-to-severe OSA. In both sets of analyses, our study showed high specificity and positive predictive values, while both sensitivity and negative predictive values were found to be low. Thus, although our study could not reliably rule out the diagnosis of OSA, the predictive values of ODI to rule in the presence of OSA were acceptable (100%). Similarly, Ryan et al. conducted a study in which the sensitivity of oximetry was reported as 31% and negative predictive value was 63%, while specificity and positive predictive values were 100% [4].

We analyzed our data with the ROC curve to see if a variable cut-off value for ODI would provide better predictive accuracy. For the AHI>5 events/hour level, the sensitivity improved to 93.5% but the negative predictive value was still low at 0.55. This was achieved at an optimal ODI of 1.2.

For the AHI≥15 events/hour level, while the sensitivity was 98% and the negative predictive value was 0.9, the specificity was reduced to 69%. At this level, the optimal ODI as determined by the AUC of the ROC curve was 1.4.

Thus, it is evident that although oximetry using type 4 equipment can say which patients definitely are suffering from OSA, it becomes difficult to rule out the disease unless the cut-off values for ODI to rule out are lowered considerably. Therefore, we find that overnight oximetry obviates the need for diagnostic PSG, helps in reducing the delays in treating the patients, and also prioritizes patients for urgent evaluation. Additionally, it should be noted that oximetry helps to find the hypoxic stress in these patients in whom long-term consequences like pulmonary hypertension and sympathetic over-stimulation can develop.

This study was limited by a reduction in the number of participating patients as the estimated sample size could not be achieved due to the ongoing COVID-19 pandemic. Future studies are required with a larger sample size. Validation and repeated calibration of the equipment used is also recommended.

## Conclusions

Overnight oximetry has high specificity and an acceptable correlation with AHI obtained by level 1 PSG in patients with a high pre-test probability of OSA. It improves the overall diagnostic accuracy of OSA and can be reliably used as a screening tool. Ruling out OSA in patients should, however, be dealt with caution.

Given the prevalence of OSA and the burden of its undiagnosed cases, especially in resource-limited settings, the ability to use portable devices like home nocturnal oximeters offers a practical solution for widespread screening and early detection. While PSG remains the gold standard, the demonstrated efficacy of home nocturnal oximetry underscores its potential role in broadening access to diagnostic services and improving patient outcomes through early intervention.

## Appendices

**Table 7:** Screening questionnaires - ESS and STOP-Bang ESS: Epworth sleepiness scale; OSA: Obstructive sleep apnea; STOP-Bang: Snoring, tiredness, observed apnea, high blood pressure, body mass index, age, neck circumference, and male gender; BMI: Body mass index; TV: Television

Screening Questionnaire		
Epworth Sleepiness Scale		
Likelihood of falling asleep in the following situation:		
Sitting and reading		
Watching TV		
Sitting inactive in a public place (e.g., a theatre or a meeting)		
As a passenger in a car for an hour without a break		
Lying down to rest in the afternoon when circumstances permit		
Sitting and talking to someone		
Sitting quietly after lunch without alcohol		
In a car, while stopped for a few minutes in traffic		
0 - No chance of dozing, 1 - Slight chance of dozing, 2 - Moderate chance of dozing, 3 - High chance of dozing		
ESS score =		
STOP-Bang Questionnaire		
STOP		
Do you SNORE loudly (loud enough to be heard through closed doors)?	Yes/No	
Do you often feel TIRED, fatigued, or sleepy during the daytime?	Yes/No	
Has anyone OBSERVED you stop breathing during your sleep?	Yes/No	
Do you have or are you being treated for high blood PRESSURE?	Yes/No	
Bang		
BMI more than 35 kg/m^2^?	Yes/No	
AGE over 50 years old?	Yes/No	
NECK circumference >16 inches (40 cm)?	Yes/No	
GENDER: Male?	Yes/No	
High risk of OSA: Yes 5-8, fIntermediate risk of OSA: Yes 3-4, Low risk of OSA: Yes 0-2		
STOP-Bang score =		
